# Treatment with Human Amniotic Suspension Allograft Improves Tendon Healing in a Rat Model of Collagenase-Induced Tendinopathy

**DOI:** 10.3390/cells8111411

**Published:** 2019-11-08

**Authors:** Laura de Girolamo, Luiz Felipe Morlin Ambra, Carlotta Perucca Orfei, John P. McQuilling, Kelly A. Kimmerling, Katie C. Mowry, Kimberly A. Johnson, Amy T. Phan, Jessica L. Whited, Andreas H. Gomoll

**Affiliations:** 1 IRCCS Istituto Ortopedico Galeazzi, Via R. Galeazzi 4, 20161 Milan, Italy; laura.degirolamo@grupposandonato.it; 2 University Hospital São Paulo, Av. Prof. Lineu Prestes, 2565-Butantã, São Paulo, SP 05508-000, Brazil; felipeambra71@gmail.com; 3 Organogenesis, 2641 Rocky Ridge Lane, Birmingham, AL 35216, USA; jmcquilling@organo.com (J.P.M.); kkimmerling@organo.com (K.A.K.); kmowry@organo.com (K.C.M.); 4 Harvard Medical School, the Harvard Stem Cell Institute, and Department of Orthopedic Surgery, Brigham and Women’s Hospital, 7 Divinity Avenue, Cambridge, MA 02138, USA; kajohnson17@gmail.com (K.A.J.); amy_phan@alumni.brown.edu (A.T.P.); jwhited@partners.org (J.L.W.); 5 Hospital for Special Surgery, 535 East 70th Street, New York, NY 10021, USA

**Keywords:** tendinopathy, amniotic membrane, collagenase, Achilles tendon, regenerative medicine

## Abstract

Treatment of tendon injuries is challenging, with neither conservative nor surgical approaches providing full recovery. Placental-derived tissues represent a promising tool for the treatment of tendon injuries. In this study, human amniotic suspension allograft (ASA) was investigated in a pre-clinical model of Achilles tendinopathy. Collagenase type I was injected in the right hind limb of Sprague Dawley rats to induce disease. Contralateral tendons were either left untreated or injected with saline as controls. Seven days following induction, tendons were injected with saline, ASA, or left untreated. Rats were sacrificed 14 and 28 days post-treatment. Histological and biomechanical analysis of tendons was completed. Fourteen days after ASA injection, improved fiber alignment and reduced cell density demonstrated improvement in degenerated tendons. Twenty-eight days post-treatment, tendons in all treatment groups showed fewer signs of degeneration, which is consistent with normal tendon healing. No statistically significant differences in histological or biomechanical analyses were observed between treatment groups at 28 days independent of the treatment they received. In this study, ASA treatment was safe, well-tolerated, and resulted in a widespread improvement of the tissue. The results of this study provide preliminary insights regarding the potential use of ASA for the treatment of Achilles tendinopathy.

## 1. Introduction

The poor intrinsic regenerative potential of tendons leads to repaired tissue that is consistently different from the native one; this tissue is characterized by altered matrix deposition, especially in terms of collagen composition and fibril alignment [1]. The lack of effective conservative treatments for chronic tendon disorders has led to the investigation and development of alternative approaches to improve outcomes. Among them, cell-based approaches appear promising, especially the use of mesenchymal stem cells (MSCs) from either adipose tissue (ASCs), bone marrow (BMSCs), platelet-rich plasma (PRP), or tendon-derived stem/progenitor cells (TSPCs) [2,3,4]. However, these autologous cell sources result in donor site morbidity and, thus, a longer and more uncomfortable surgical procedure and recovery for patients. Alternatively, the use of an allogenic source would allow physicians to overcome these limitations while maintaining the cell therapeutic effects. Several studies in the literature highlight the potential of amniotic membrane and amniotic fluid for a variety of orthopedic indications, as they are sources of collagen, extracellular matrix (ECM) proteins, growth and reparative factors, and stem cells [5,6,7].

Human placental membranes are made up of the amnion layer (the inner layer closest to the fetus) and the chorion layer (the outer protective layer). Amniotic fluid resides within the amniotic sac, communicating with the amnion layer, and contains cells, proteins, carbohydrates, lipids, phospholipids, urea, electrolytes, and water [8]. Together, the fluid and membrane components contain many growth factors and cytokines that aid in the development of the growing fetus, providing an anti-inflammatory environment that serves to protect the unborn child [8,9]. Human amniotic suspension allograft (ASA) contains particulated human amniotic membrane (HAM) and cells derived from the amniotic fluid. Placental-derived tissues contain several regenerative and anti-inflammatory molecules, including insulin-like growth factor I (IGF-I), platelet-derived growth factor BB (PDGF-BB), transforming growth factor beta 1 (TGF-β1), interleukin-1 receptor antagonist (IL-1Ra), and tissue inhibitors of matrix metalloproteinases (TIMPs) [10,11,12,13,14].

Cells and tissue from the placenta have been used in several orthopaedic studies in vitro and in vivo with promising results. Several studies have examined the use of HAM and human amniotic fluid cells (HAFCs) to promote tendon healing in preclinical models [9,11,15,16,17,18]. Barboni et al. applied amniotic epithelial cells (AECs) at the site of defects in sheep Achilles tendons, and demonstrated improved mechanical and structural healing compared to groups that didn’t receive AECs [15]. In a rabbit model of flexor digitorum fibularis repair, human amniotic fluid (HAF) was injected into the surgical site along with sheath excision; HAF-treated animals showed higher tensile load values and less adhesions compared to groups not treated with HAF [18].

Additionally, several groups have evaluated the effect of cytokines and growth factors on tendon repair [19,20,21]. Two studies used conditioned media from human amniotic mesenchymal stem cells (AMSCs) or amniotic membrane progenitor cells (AMPCs) at the site of Achilles tendon transection in rats; they found increased collagen production and biomechanical properties following treatment [20,21]. Placental membrane particulate containing growth factors and ECM has also been evaluated. Clinically, Gellhorn et al. used a dehydrated human amnion/chorion membrane allograft injection for patients with chronic tendinopathy or arthropathy. This study found that 91% of patients had 30% or greater improvement of pain at 3 months following injection, and activities of daily living (ADL) functional impairment scores improved/decreased from 6.8 to 2.0 [22]. In a different clinical application, the same ASA evaluated in the current study was also used in a six-patient pilot study evaluating its safety and efficacy as a treatment for symptomatic knee osteoarthritis; no adverse effects were reported and a full, randomized, multicenter study is underway [7].

These preclinical and clinical studies provide rationale supporting the use of ASA as a potential treatment for broader inflammatory conditions. In this study, we evaluated the use of ASA in a rat Achilles collagenase-induced tendinopathy model and hypothesized that ASA would result in improved tendon healing as examined through histological and biomechanical analysis compared to untreated groups. 

## 2. Materials and Methods

### 2.1. Ethics Statement

The study protocol was approved by the Brigham and Women’s Hospital Institutional Animal Care and Use Committee (Protocol # 2016N000527). The protocol as submitted and reviewed conformed to the United States Department of Agriculture Animal Welfare Act, Public Health Service Policy on Humane Care and Use of Laboratory Animals, the Institute for Laboratory Animal Research Guide for the Care and Use of Laboratory Animals, and other applicable laws and regulations. The animals were regularly evaluated by a certified veterinarian responsible for health monitoring, animal welfare supervision, experimental protocols and procedure revision. All surgeries were performed under general anesthesia, and all efforts were made to minimize suffering.

### 2.2. Study Design

**Figure 1:**
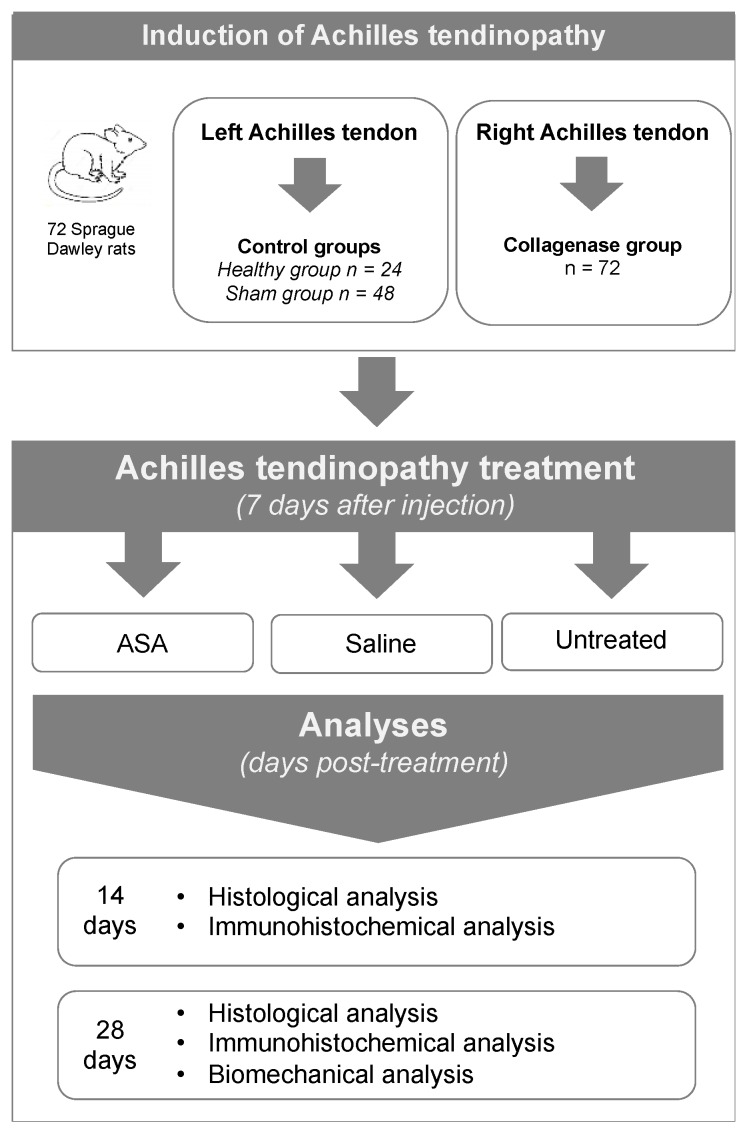
Experimental design for induction of Achilles tendinopathy. 72 Sprague Dawley rats underwent collagenase-induced tendinopathy in their right Achilles tendon. 24 of these rats had untouched left Achilles tendons (healthy controls), while 48 of these rats had dry needling on their left Achilles tendons (sham controls). Seven days following collagenase injection, rats were either treated with saline (vehicle control), amniotic suspension allograft (ASA), or left untreated. Fourteen days post-treatment, tendons were collected for histological analysis and immunohistochemical analysis. Twenty-eight days post-treatment, tendons were collected for histological analysis, immunohistochemical analysis, and biomechanical analysis.

Seventy-two (72) Sprague Dawley rats (8–9 weeks old) were used in this study. During the procedures, the rats were anesthetized via inhalation of isoflurane (3%; Merial, Duluth, Georgia, USA). The pathology induction phase consisted of the generation of Achilles tendinopathy by intra-tendinous injection of collagenase type I in the right limb of each animal (n = 72) according to a previously published model [23]. The contralateral Achilles tendon was used as a non-injured control (Figure 1). The intervention procedures were performed 7 days after disease induction, and animals were sacrificed 14 or 28 days post-treatment. Tendons were harvested and evaluated by histological and immunohistochemical analyses at both 14 and 28 days post-treatment, while the biomechanical properties of tendons in terms of tensile strength were evaluated at 28 days post-treatment only (Figure 1). 

### 2.3. In Vivo Procedures

#### 2.3.1. Achilles Tendinopathy Induction

Achilles tendinopathy was induced by injecting 3 mg/mL of collagenase type I (Worthington Lakewood, NJ, USA, 185 IU/mg) under ultrasound guidance in the right Achilles tendon of each animal with a 30 G needle (collagenase group, n = 72). The contralateral Achilles tendon was treated with either dry needling (sham group, n = 48) or left untreated (healthy group, n = 24).

#### 2.3.2. Amniotic Suspension Allograft (ASA) Preparation 

For this study and to minimize donor-to-donor variability, ASA from 3 donors was combined. ASA was stored at −80°C until use. Prior to use, ASA was thawed and diluted at a 1:1 ratio with sterile normal saline and kept on ice for no longer than one hour prior to use.

#### 2.3.3. Achilles Tendinopathy Treatment with ASA 

Seven days following pathology induction with collagenase, the right hind legs of each rat were treated with 40 µL of either ASA (collagenase + ASA group, n = 24), saline (collagenase + saline group, n = 24), or untreated (collagenase group, n = 24). Treatments at 7 days of either saline or ASA were injected under ultrasound guidance using a 30 G needle. The left hind leg tendon (without collagenase injection) received identical treatment injections at 7 days as reported above: ASA (sham + ASA group, n = 24), saline (sham + saline group, n = 24) or untreated (healthy group, n = 24) (Table 1). 

#### 2.3.4. Animal Care and Euthanasia

After each procedure, tramadol (1 mg/kg) was administered orally to the animals for the first 24–48 h every 24 h, and then as needed for pain. The rats were monitored by the investigators daily for the first week after injection, then twice a week for the remaining follow-up period for behavior, food and water intake, and any signs of self-trauma. Additionally, technicians at the animal care facility provided routine care and supervision outside that from the investigators. Animals were euthanized at 14 (n = 24) or 28 days (n = 48) post-treatment by CO_2_ inhalation to harvest the Achilles tendons for analyses.

### 2.4. Histological and Immunohistochemical (IHC) Analyses

Specimens were fixed in 4% paraformaldehyde (PFA) for 24 h at room temperature. Fixed samples were then washed in phosphate buffered saline (PBS) solution and soaked in sucrose solutions at increasing concentrations (10%, 20% and 30%) and left overnight in 30% sucrose at room temperature. The samples were then frozen in liquid nitrogen, embedded in Optimal Cutting Temperature (OCT) compound, and kept at −80 °C until sectioning. Samples were cut into 8 µm-thick serial sections and stained with Hematoxylin and Eosin (H&E) to evaluate tendon morphology. Each sample was evaluated by three blinded observers according to the semi-quantitative grading score (0 = best score, 3 = worst score) previously used [24,25] (Table 2), with a total possible score of 18. The parameters examined included fiber structure, fiber arrangement, rounding of nuclei, inflammation, increased vascularity, and cell density.

Each sample was also stained using IHC for collagen type I and III. Briefly, the tissue sections were incubated overnight at 4 °C with the following primary antibodies diluted in PBS-BT buffer (PBS buffer supplemented with 1% bovine serum albumin and 0.5% Triton™ X-100): rabbit polyclonal anti-collagen I, 1:200 (ab34710, Abcam, Cambridge, MA, USA) and rabbit polyclonal anti-collagen III, 1:500 (ab7778, Abcam, Cambridge, MA, USA). For negative controls, the primary antibody was omitted from the PBS-BT buffer that was applied to respective sections. Afterwards, all sections were washed with PBS buffer and incubated for one hour with UltraPolymer goat anti rabbit–horseradish peroxidase (HRP) (BioVision, Milpitas, CA, USA). 

The same IHC protocol described above was used for the detection of human tissue inside sections. Human nuclei (within rat tendons) were stained by an overnight incubation at 4 °C with anti-human nuclei clone 3E1.3 (MAB 4383, MilliporeSigma, Burlington, MA, USA) diluted 1:200 in PBS-BT buffer. Detection was performed by UltraPolymer Goat anti mouse–HRP (BioVision, Milpitas, CA, USA). For all IHC protocols, diaminobenzidine (ImmPACT DAB peroxidase, Vector Labs, Burlingame, CA, USA) was used as a chromogenic substrate of the peroxidase reaction.

### 2.5. Biomechanical Testing 

Biomechanical testing was conducted on a total of 48 tendons (8 tendons per group, Table 1) at 28 days post-treatment. The tendons were left at their bony attachment site distally, and the triceps surae muscle was transected through the muscle belly. The samples were kept at −80 °C, thawed at room temperature, and wrapped in PBS-soaked gauze to prevent drying out. The tendons were secured to the mechanical test machine with the samples aligned in the direction of its pull by gripping the muscle belly proximally and the bone insertion distally. The eXpert 5603 universal testing machine (Admet, MA, USA) was used. After the sample was secured, the tendon was placed under tensile stress at a constant rate of 10.0 mm/min until failure. Failure was defined as the maximum load after the first drop in load tension. Mechanical load failure was calculated and expressed in Newtons.

### 2.6. Statistical Analyses 

A Shapiro-Wilk test was used to assess the distribution of data. Experimental groups and time points were compared and analyzed by Mann-Whitney U test (GraphPad Prism v5.00 Software, La Jolla, CA, USA). Data are expressed as median values with range. Values of *p* < 0.05 were considered statistically significant. 

## 3. Results

### 3.1. Histopathological Findings and Score Analyses 

**Figure 2:**
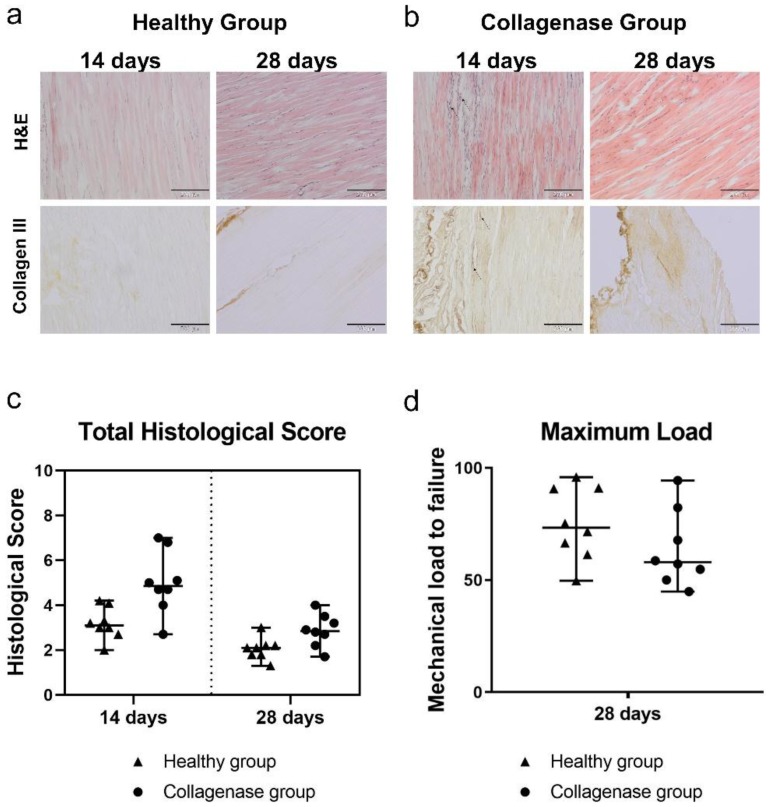
Histological and biomechanical analysis for the untreated control groups. Hematoxylin & Eosin (H&E) staining and Collagen III immunohistochemical (IHC) staining of the (**a**) healthy group (untreated/untreated) and (**b**) collagenase group (collagenase/untreated) at both 14 and 28 days post-treatment. Scale bar indicates 200 μm. (**c**) Total histological score for tendons in the healthy and collagenase group at 14 and 28 days post-treatment. Median with range reported. (**d**) Maximum load for tendons in the healthy and collagenase group at 28 days post-treatment. Median with range reported.

In the healthy control group, the tendons were hypocellular, showing a normal collagen fiber alignment with tenocytes arranged parallel to the fibers. No adipose tissue degeneration, inflammatory cells, or neovascularized tissue portions were observed in the healthy control group (Figure 2a). At 14 days, injection of 3 mg/mL collagenase type I elicited a loss of the typical macroscopic structure of the tendon with augmented cell density (mainly with a rounded morphology), disorganization of fiber arrangement, and areas of neovascularization were present (Figure 2b). Qualitatively, increased adipose tissue was also observed. At 14 days, the total histological score of the collagenase group was worse (median 4.9, range 2.7–7.0) than those of the healthy group (median 3.1, range 2.0–4.2), as expected (Figure 2c). 

Compared to 14 days, at 28 days after disease induction, tendons appeared less degenerated with a more regular fiber alignment, along with less tissue deterioration and a reduced presence of inflammatory cells, consistent with physiological tendon healing (Figure 2a,b). The median values of the histological scores in the collagenase-treated and healthy tendons were 2.9 (range 1.7–4.0) and 2.1 (range 1.3–3.0), respectively (Figure 2c). The altered structure of collagenase-treated tendons was associated with an increase in collagen III deposition (Figure 2b) that was found mainly localized in the most peripheral portion of the tissue in proximity of the peritenon. The healthy tendons did not demonstrate any deposition of this molecule at either time points (Figure 2a), whereas collagen I was consistently expressed without relevant differences among all the groups. Biomechanical testing showed that at 28 days post-treatment, the mechanical load to failure was lower in the collagenase group (median 57.93 N, range 44.87–94.40) compared with healthy controls (median 73.41 N, range 49.68–95.89) (Figure 2d), although not statistically significantly. 

**Figure 3:**
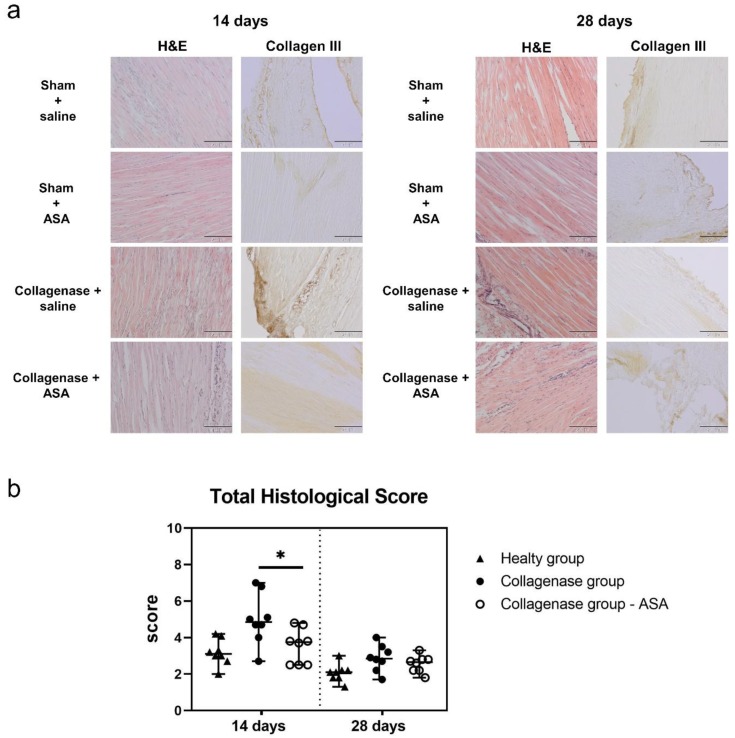
Histological analysis for the treated groups. (**a**) Hematoxylin & Eosin (H&E) staining and Collagen III immunohistochemical (IHC) staining of the sham/saline, sham/amniotic suspension allograft (ASA), collagenase/saline, and collagenase/ASA groups at 14 days and 28 days post-treatment. Scale bar indicates 200 μm. (**b**) Total histological score for tendons in the Healthy group, the Collagenase group and the Collagenase group – ASA at 14 and 28 days post-treatment. Median with range reported. * denotes *p* < 0.05 by Mann-Whitney U test.

By 14 days following treatment, the tendons treated with collagenase and then subsequently injected with ASA showed an improvement in the macroscopic tissue structure in term of fiber organization, cell density and fatty deposit formation when compared with the collagenase + saline group (Figure 3a). At 14 days, the histological score of the collagenase + ASA group showed a significant improvement in comparison to the collagenase + untreated group (*p* < 0.05, Collagenase group total score median value of 4.9 with range 2.7–7.0 and Collagenase group – ASA total score median value of 3.8 with range 2.5–4.8). At 28 days, the tendons in both treatment groups showed less signs of degeneration with respect to the previous time point, consistent with expected tendon healing (Figure 3a,b). Qualitatively, fiber alignment, as well as the cell density and the presence of inflammatory cells, appeared improved in the collagenase + ASA group, which was the only group that was not significantly different from the healthy tendons (Healthy group total score median value 2.1, range 1.3–3.0; Collagenase group – ASA total score median value, 2.7 with range 1.8–3.3 Supplemental Table S1).

**Figure 4:**
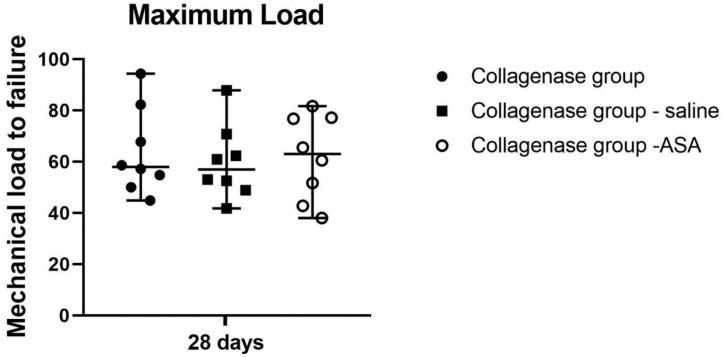
Biomechanical analysis for the treated groups. Maximum load for tendons in the collagenase/untreated, collagenase/saline, and collagenase/amniotic suspension allograft (ASA). Median with range reported.

Biomechanical testing at 28 days yielded a trend towards better results in the ASA group without statistically significant differences between groups (maximum loads median values: Collagenase group 57.93 N, range 44.87–94.40, Collagenase group + saline 60.94, range 41.79–87.91, Collagenase group – ASA, 63.04, range 38.06–81.73, Figure 4). 

### 3.2. Qualitative Observations Of Retention of ASA 

At both time points assayed following ASA treatment, all samples were stained with anti-human nuclei clone 3E1.3 to identify any remaining ASA present within the tendon tissue. The IHC analysis confirmed the presence of residual human nuclei at both time points within rat tendons. These cells were found both in collagenase-treated samples and in the sham groups, although more abundant cells were observed in the former. 

**Figure 5:**
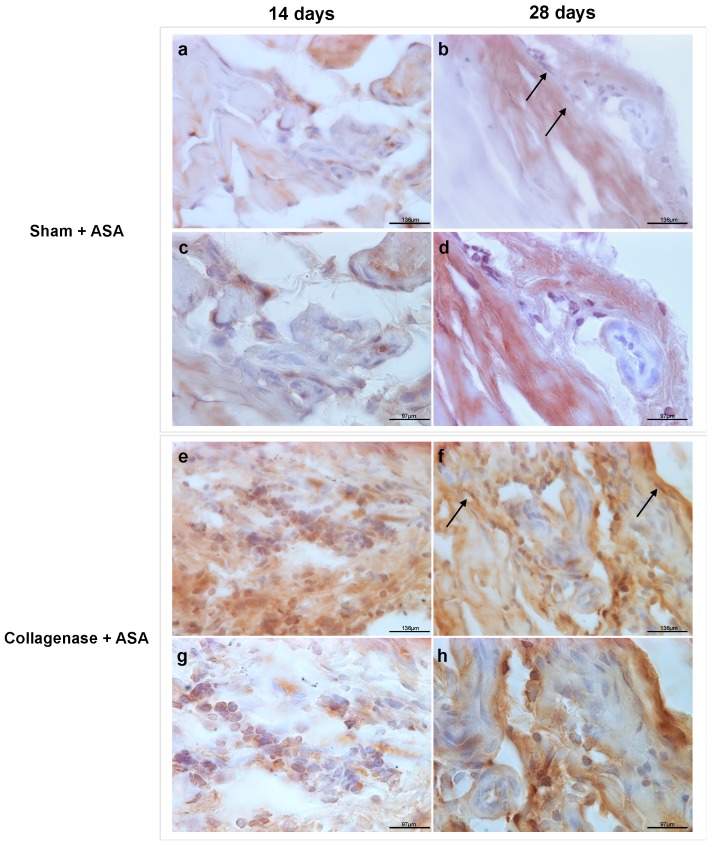
Anti-human nuclei clone 3E1.3 immunohistochemical (IHC) staining in the amniotic suspension allograft (ASA) treated groups for the presence of ASA at each time point. For 14 days, (**a**) and (**c**) denote sham/ASA, while (**e**) and (**g**) denote collagenase/ASA. For 28 days, (**b**) and (**d**) denote sham/ASA, while (**f**) and (**h**) denote collagenase/ASA. Scale bars for (**a**,**b**,**e**,**f**) indicates 136 μm, while scale bars for (**c**,**d**,**g**,**h**) indicates 97 μm. Black arrows indicate postively-stained cells, indicating the retention of ASA in the tendon at the specific time point.

Positive staining for human nuclei were specifically detectable in the proximity of the peripheral portion of the tendon, near the peritenon. At 14 days, positively stained portions appeared aggregated (Figure 5a,c,e,g), whereas at 28 days they appeared more integrated with tendon fibers (Figure 5b,d,f,h).

## 4. Discussion

In this study, the potential therapeutic effect of ASA was tested in an established model of chronic Achilles tendinopathy assessing tendon morphological features, including fiber alignment, cell density, presence or absence of an inflammatory reaction, neovascularization process, adipose tissue infiltration, and matrix composition. In general, the treatment with ASA in sham animals and those that had collagenase disease induction demonstrated safety and was well tolerated by the animals; no adverse or inflammatory reactions were observed, and ASA did not provoke any deterioration in terms of fiber organization, fatty deposit formation or other cell morphology alteration. Moreover, administration of ASA resulted in improved healing of the tissue at 14 days, as demonstrated by better histological scores and a lower presence of collagen type III with respect to animals that were treated with saline only or untreated controls.

Even though an animal model that can completely reproduce human tendinopathy has not yet been developed, the rat represents the most popular species to model Achilles tendinopathy because the size of its Achilles tendon allows for robust histological and biomechanical testing and analysis. For these reasons, the rat model of Achilles tendinopathy has been extensively used in preclinical research due to similar limb anatomy [26,27] and the genetic homology to humans [28]. One of the most common techniques to develop rodent models of tendinopathy is based on mechanical overuse; however, this model is not completely accepted due to the lack of reproducibility and resulting inflammation that does not mimic clinical human tendinopathy [27,29]. Among the chemically-induced tendinopathy models, it has been shown that collagenase type I can provoke collagen fiber disruption and changes in biochemical and biomechanical features of the tendon, closely resembling the main histopathological characteristics and functional impairments of human tendinopathy [26,27,30]. Thus, this injection model is recognized as a valid approach to induce and study the development of this pathology [31]. Although different concentrations of collagenase have been tested in rats, the accepted injection range is 1–3 mg/mL [32,33]. In a previous study, we observed that the most severe grade of tendinopathy in the rat Achilles tendon occurs about 10 to 14 days following collagenase injection [23]. At this time point, the tendon tissue was characterized by a marked misalignment of fibers, increased number of rounded cells, and more abundant adipose tissue infiltration and neo-vessel formation, all features of a pathological tendon [34]. Our choice to use the rat Achilles tendon with collagenase injury to evaluate ASA treatment was based on solid guidance by the existing literature. 

In the present study, as expected, histological scores were always higher (worse) for pathological tendons compared to healthy tendons. Given the pathophysiological process of tissue repair, degeneration due to disease induction with collagenase was worse at 14 days than at 28 days. However, the long-term expression of collagen type III in the pathological tendons at 28 days confirms that the spontaneous healing process generally leads to the production of fibrotic tissue [35]; fibrotic tissue has been associated with development of tissue adhesions, formation of scar tissue, and occasional ectopic bone formation [36]. The biomechanical analysis also confirmed this aspect, since it revealed a lower, albeit not significant, maximum load in the pathological tendons compared to the healthy tendons [37,38,39]. 

The ASA treatment was well tolerated, and overall, this study provided important evidence regarding the retention of ASA within the tendon tissue after treatment. At 14 days, ASA was present specifically in the peripheral area of the tissue, whereas at 28 days, ASA was more closely associated with the tendon fibers. Additionally, injection with ASA showed no signs of adverse reactions, confirming the low immunogenicity of placental-derived tissue. Retention of ASA within the tissue for an extended length of time, along with improvements in tendon repair due to ASA treatment, suggest a potential benefit of ASA for tendon healing. 

After the injection of collagenase type I, the administration of ASA led to significant improvements in terms of fiber alignment and ECM restoration with greater physiologic cellularity at two weeks. This evidence agrees with results demonstrated by Demirkan et al., who evaluated the effects of amniotic membrane in an ovine model of flexor tendon excision; they observed relevant enhancement of tendon structure remodeling and a marked decrease of tendon adhesions [40]. Barboni et al. reported treatment with human amniotic epithelial cells (hAEC) resulted in better structural repair in an Achilles tendinopathy sheep model [15]. Additionally, Philip et al. showed that the administration of amnion-derived multipotent progenitor cells (AMPCs) resulted in a significant increase in the mechanical strength of the tissue compared to controls in a rat Achilles tendon transection model [21]. In our current study, only a trend without significant differences was observed in the biomechanical evaluation. This may be due to the sample size in each group or the spontaneous tendon repair in all groups could have masked a potential benefit mediated by the ASA at 28 days post-treatment. Performing the same analysis at an earlier time point may provide more conclusive results. However, the outcomes of this study clearly revealed enhanced tissue repair following treatment with ASA. 

One limitation of the study is that we administered ASA only once; we did not assess whether multiple treatments would provide an additional benefit in this model. Another limitation of the study is the adoption of an ultrasound-guided injection technique for induction of tendinopathy and subsequent treatments. While an open-surgery approach to expose the tendon would have potentially provided improved precision, it would have been significantly more invasive and was not pursued because rats were treated bilaterally and underwent multiple procedures. The presence of human tissue inside the rat Achilles tendon, however, demonstrates the overall success of our approach.

## 5. Conclusions

In conclusion, the results of this study support the hypothesis that ASA has potential beneficial effects for the treatment of Achilles tendinopathy. However, further studies are needed to determine the optimal dosing and injection strategy to provide the best outcomes in a larger animal model. Additionally, clinical studies should be completed to validate these effects in a human population.

## Figures and Tables

**Table 1:** Model Overview and Treatment Groups.

Right Achilles Tendon (n = 72)		14 Days Post-Treatment	28 Days Post-Treatment	
Induction of the pathology (day 0)	Treatment of the pathology (day 7)	Histological analysis (n)	Histological analysis (n)	Biomechanical analysis (n)
Collagenase group	Untreated	8	8	8
Collagenase group	ASA	8	8	8
Collagenase group	Saline	8	8	8

**Table 2:** Grading system for the histological evaluation of tendons (H&E).

Score	Fiber Structure and Arrangement	Cell Density	Cell Appearance	Inflammatory Cell Inflammation	Neovascularization	Fatty Deposits
0	Normal: continuous, parallel collagen fibers	Normal	Spindle-shape cells	<10%	Normal presence of vascular bundles	Absence of lipid vacuoles
1	Slightly abnormal: partially disorganized and fragmented fibers	Slightly increased	Slightly rounded cells	10–20%	Slight increase of vascular bundles	Slight increase of lipid vacuoles
2	Abnormal: moderately disorganized, fragmented, crossed, and wavy fibers	Moderately increased	Moderately rounded cells	20–30%	Moderate increase of vascular bundles	Moderate increase of lipid vacuoles
3	Markedly abnormal: total disorganized and non-identifiable fiber pattern	Markedly increased	Markedly rounded cells	>30%	Marked increase of vascular bundles	Marked increase of lipid vacuoles

## Supplementary Materials

The following are available online at https://www.mdpi.com/2073-4409/8/11/1411/s1, Table S1: Raw scores for histological evaluation of tendons on H&E stained sections.
