# Supplementary material for: Treatment with Human Amniotic Suspension Allograft Improves Tendon Healing in a Rat Model of Collagenase-Induced Tendinopathy
Source: Cells. 2019 Nov 8;8(11):1411. doi: 10.3390/cells8111411 (PMC6912389; doi:10.3390/cells8111411)
Supplement: Supplementary file 1 [file cells-08-01411-s001.pdf]

Supplemental Table 1: Raw scores for the histological evaluation of tendons on Hematoxylin & Eosin (H&E) stained sections.

		Fiber Structure	Cell Density	Cell Morphology	Inflammation	Neo-vascularizatio n	Adipose Tissue Degeneration	TOTAL SCORE
**Control**	**14 days**	0.2 ± 0.4	1.1 ± 0.5	0.9 ± 0.5	0.5 ± 0.4	0.3 ± 0.3	0.1 ± 0.3	**3.1 ± 0.7**
	**28 days**	0.3 ± 0.3	0.7 ± 0.3	0.6 ± 0.5	0.5 ± 0.2	0.0 ± 0.0	0.1 ± 0.2	**2.1 ± 0.7**
**Sham + Saline**	**14 days**	0.3 ± 0.4	1.5 ± 0.8	0.8 ± 0.6	0.7 ± 0.5	0.2 ± 0.4	0.2 ± 0.4	**3.7 ± 0.5**
	**28 days**	0.3 ± 0.2	0.9 ± 0.4	1.0 ± 0.5	0.6 ± 0.5	0.0 ± 0.1	0.2 ± 0.4	**2.9 ± 1.0**
**Collagenase**	**14 days**	0.7 ± 0.4	1.7 ± 0.7	0.9 ± 0.6	0.3 ± 0.5	0.5 ± 0.5	0.6 ± 0.7	**5.0 ± 1.7**
	**28 days**	0.3 ± 0.3	0.8 ± 0.5	1.0 ± 0.4	0.5 ± 0.3	0.0 ± 0.1	0.3 ± 0.4	**2.9 ± 0.9**
**Collagenase + Saline**	**14 days**	0.5 ± 0.5	1.1 ± 0.5	1.1 ± 0.5	0.8 ± 0.7	0.3 ± 0.4	0.5 ± 0.7	**4.2 ± 1.6**
	**28 days**	0.2 ± 0.3	1.1 ± 0.4	1.3 ± 0.7	0.6 ± 0.3	0.0 ± 0.0	0.0 ± 0.0	**3.1 ± 0.8**
**Sham + ASA**	**14 days**	0.3 ± 0.4	1.2 ± 0.6	1.2 ± 0.6	0.7 ± 0.5	0.3 ± 0.5	0.3 ± 0.5	**4.0 ± 0.6**
	**28 days**	0.2 ± 0.3	0.8 ± 0.5	1.0 ± 0.6	0.4 ± 0.4	0.1 ± 0.2	0.1 ± 0.3	**2.5 ± 1.1**
**Collagenase + ASA**	**14 days**	0.5 ± 0.5	1.3 ± 0.7	0.9 ± 0.5	0.9 ± 0.5	0.1 ± 0.2	0.0 ± 0.1	**3.7 ± 1.0**
	**28 days**	0.3 ± 0.3	0.8 ± 0.4	0.8 ± 0.5	04 ± 0.3	0.1 ± 0.2	0.1 ± 0.4	**2.6 ± 0.5**
